# Polydatin accelerates osteoporotic bone repair by inducing the osteogenesis-angiogenesis coupling of bone marrow mesenchymal stem cells via the PI3K/AKT/GSK-3β/β-catenin pathway

**DOI:** 10.1097/JS9.0000000000002075

**Published:** 2024-09-06

**Authors:** Chunhao Zhou, Guanyu Hu, Yikai Li, Sheng Zheng

**Affiliations:** a Department of Orthopedics, Division of Spine Surgery, Nanfang Hospital, Southern Medical University; b Department of Traditional Chinese Orthopedics and Traumatology, Center for Orthopedic Surgery, The Third Affiliated Hospital of Southern Medical University, Guangzhou, People’s Republic of China

**Keywords:** bone marrow mesenchymal stem cells, osteogenesis-angiogenesis coupling, osteoporotic bone repair, PI3K/AKT/GSK-3β/β-catenin, polydatin

## Abstract

**Background:**

Polydatin (POL), a natural stilbenoid, has multiple pharmacological activities. However, its effect on osteoporotic bone defects has not yet been examined. This study was designed to explore the unknown role of POL on osteoporotic bone repair.

**Methods:**

The effect of POL on osteogenesis and angiogenesis were investigated firstly. Then a series of angiogenesis-related assays were carried out to explore the relationship between osteogenesis and angiogenesis of POL, and the underlying mechanism was further explored. Whereafter, ovariectomy-induced osteoporosis rats with bone defect were treated with POL or placebo, the imageological and histological examinations were conducted to assess the effect of POL on osteoporotic bone repair.

**Results:**

The moderate concentrations (1 μM and 10 μM) of POL enhanced the osteogenesis of bone marrow mesenchymal stem cells (BMSCs) and elevated the expression of angiogenic-specific markers. Further research found that POL-induced human umbilical vein endothelial cells migration and tube formation through the osteogenesis-angiogenesis coupling of BMSCs, and the POL-induced osteogenesis-angiogenesis coupling was reversed after co-cultured with LY294002. Mechanistically, this was conducted via activating PI3K/AKT/GSK-3β/β-catenin pathway. After that, using the osteoporotic bone defect rat model, the authors, observed that POL facilitated osteoporotic bone repair through enhancing osteogenesis and CD31^hi^EMCN^hi^ type H-positive vessels formation via the PI3K/AKT/GSK-3β/β-catenin pathway.

**Conclusion:**

The data above indicated that POL could accelerate osteoporotic bone repair by inducing the osteogenesis-angiogenesis coupling of BMSCs via the PI3K/AKT/GSK-3β/β-catenin pathway, which provided new insight and strategy for osteoporotic bone repair.

## Graphical abstract

**Figure:**
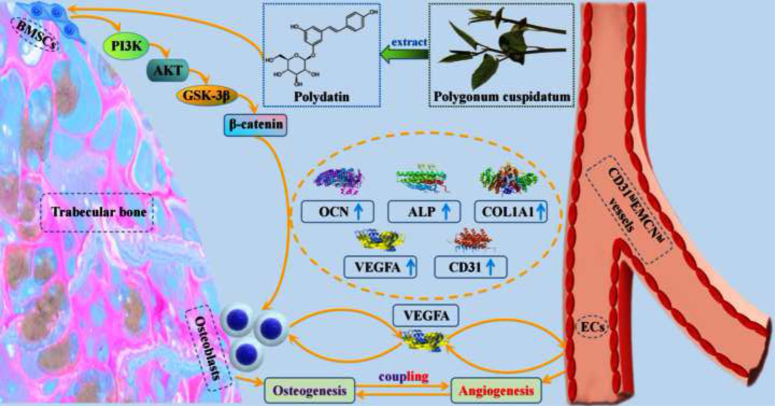

## Introduction

Highlights

- Polydatin possessed the ability to promote BMSCs-mediated angiogenesis.
- Polydatin-induced osteogenesis-angiogenesis coupling of BMSCs via activating PI3K/AKT/GSK-3β/β-catenin pathway.
- Polydatin enhanced osteogenesis and CD31^hi^EMCN^hi^ type H-positive vessels formation of bone defect area in ovariectomized rats.

Osteoporosis is the most frequent metabolic bone disease in postmenopausal women, ~30% of women in the United States are at risk for osteoporosis and about 200 million women worldwide suffer from osteoporosis^1,2^. Osteoporotic bone defect, a common but severe complication of osteoporosis which is usually secondary to the fracture or surgery, has posed a high risk of disability and mortality for osteoporotic patients. Although bone tissue itself has a relatively strong ability for bone repair, the recovery duration of osteoporotic bone defect is inevitably prolonged due to the overabundance of bone resorption and the inhibition of bone formation^3^. The surgical therapies such as allografts, autografts, and traction osteogenesis, which are already clinically available, potentially result in plenty of intraoperative or postoperative complications, and the therapy outcomes are still not very satisfactory^4^. To date, the management of osteoporotic bone defect is still an intractability issue^5^, thus there is an urgent need to explore novel therapeutic strategies for the treatment of osteoporotic bone defect.

Polydatin (POL), a natural stilbenoid extracted from the traditional plant Polygonum cuspidatum, is well known for a wide range of beneficial effects, including antioxidative, anti-inflammatory, and antitumor properties^6–8^. In recent years, studies have confirmed that POL could enhance osteogenesis and play an antiosteoporosis role^9,10^. However, the effect of POL on osteoporotic bone defect has not yet been examined. Besides osteogenesis, angiogenesis is an indispensable process for the repair of osteoporotic bone defect^11^. Regretfully, so far the definite role of POL on angiogenesis in the bone microenvironment remains obscure. It is worth noting that the osteogenesis and angiogenesis is a tightly, well-coordinated coupling process during bone repair^12^. Although POL is reported to promote osteogenic differentiation of bone marrow mesenchymal stem cells (BMSCs)^13^, its effect on coordinating osteogenesis and angiogenesis has never been revealed. Based on the above understanding, it is very meaningful to explore the relationship between osteogenesis and angiogenesis of POL during osteoporotic bone repair.

Regarding the underlying mechanism, the PI3K/AKT pathway involved in the regulation of osteogenesis and angiogenesis has been widely acknowledged^14,15^. After activation, phosphorylated PI3K activates the AKT and subsequently alters the activity of downstream targets such as mTOR, HIF-1α, and GSK3-β^16–18^. It was reported that POL exhibited notable antiosteoporotic activity via regulating β-catenin^19^. As an indispensable signaling in osteogenic BMSCs, accumulated β-catenin in cytoplasm exerts its effects through transport to the nucleus, where it regulates the expression of multiple osteogenic genes, β-catenin has been verified to be directedly regulated by GSK-3β, phosphorylated β-catenin by GSK-3β leads to ubiquitin-mediated degradation of β-catenin in the proteasome^20^. On the other hand, GSK-3β is also one of the downstream effectors of PI3K/AKT signaling, suggesting that POL may play a beneficial role during bone repair in osteoporotic bone by regulating osteogenesis and angiogenesis via the PI3K/AKT/GSK-3β/β-catenin pathway. Therefore, this study was designed to explore the unknown role and underlying mechanism of POL on osteoporotic bone repair.

## Materials and methods

### Chemicals and reagents

POL (purity ≥99.0%, Cat. No. ZES-4974S) was ordered from Zzbio Co., Ltd. (Shanghai, China). Monoclonal antibodies against CD29, CD73, CD90, CD105, and CD34 were purchased from eBioscience (Waltham, USA). Primary antibodies against osteocalcin (OCN), runt-related transcription factor 2 (RUNX2), alkaline phosphatase (ALP), collagen type I alpha 1 (COL1A1), vascular endothelial growth factor A (VEGFA), platelet endothelial cell adhesion molecule-1 (CD31), endomucin (EMCN), PI3K, phosphorylated-PI3K (p-PI3K), AKT, phosphorylated-AKT (p-AKT), GSK-3β and phosphorylated-GSK-3β (p-GSK-3β), and GAPDH were from Affinity Biosciences (Cincinnati, USA). Primary antibodies against β-catenin and phosphorylated β-catenin (p-β-catenin) were purchased from Cell Signaling Technology (Boston, USA). LY294002 was ordered from Beyotime (Shanghai, China).

### Cell counting kit-8 (CCK-8) assay

BMSCs were isolated and cultured as previously described^21^. BMSCs at passage 3 were collected and subjected to analysis on a CytoFLEX flow cytometer (Beckman Coulter, USA). For phenotypic analysis, the expression of CD29, CD73, CD90, CD105, and CD34 were evaluated. After phenotypic analysis, the CCK-8 (Cat. No. CA1210, Solarbio, China) was used to assess the effect of POL on BMSCs proliferation. BMSCs were cultured in a complete medium with different concentrations of POL (0, 0.1, 1, 10, 100 M) for days 1, 3, 5, and 7. The untreated group served as a control. At the end of the assay, each well was added with 100 μl CCK-8 solution (90 μl α-MEM + 10 μl CCK-8 reagent), and BMSCs were incubated at 37°C for 1 h. Absorbance was read on a microplate reader (Molecular Devices Co., Ltd, San Jose, CA, USA) at 450 nm.

### Colony-forming unit (CFU) assay

BMSCs at passage 3 were seeded into 96-well plates (1×10^3^ cells/well) and incubated in the presence or absence of POL for 7 days. Then, the clones were fixed with 4% paraformaldehyde and stained with 0.1% crystal violet for 20 min. Colonies containing 50 or more cells were quantified by ImageJ software (Image J 1.51, NIH, USA).

### Osteogenic differentiation assays

When BMSCs confluency reached 80%, the mediums were replaced with osteogenic medium (complete α-MEM containing 10 nM dexamethasone, 50 μM ascorbic acid, and 10 mM β-glycerophosphate). Then, different concentrations of POL (0, 1, and 10 μM) were added into the mediums. Total cellular proteins were extracted and the supernatant liquid were collected for the downstream assays. ALP activity was measured with ALP Staining Kit (Cat. No. G1481, Solarbio, China) after 7 days. Calcium mineralization was observed with alizarin red S (ARS) solution (Cat. No. G1452, Solarbio, China) after 21 days.

### Quantitative real-time PCR (qRT-PCR)

Total RNA was extracted with an RNA Purification Kit and reverse transcription was performed with a cDNA Reverse Transcription Kit. qRT-PCR was performed using SYBR Green qPCR Master Mix. Relative gene expression was calculated using the 2^−ΔΔCT^ method, and GAPDH was used as a reference for normalization. The qPCR primers were listed in Table S1 (Supplemental Digital Content 2, http://links.lww.com/JS9/D396).

### Western blot analysis

Total protein was extracted using the RIPA lysis buffer with protease inhibitor and protein phosphatase inhibitor on the ice. Then, the cell lysates were collected and received ultrasonication for 10 min. After 15 min of centrifugation (4°C, 12 000 rpm), the protein concentration was measured with a BCA Protein Assay Kit. Equal amounts of protein (30 μg) were subjected to 10% SDS-PAGE and blotted onto PVDF membranes (PVDF, Millipore, USA). After being blocked with QuickBlock Blocking Buffer for western blot, the membranes were incubated with primary antibodies against OCN (Cat. No. DF12303, 1:1000), RUNX2 (Cat. No. AF5186, 1:1000), ALP (Cat. No. DF6225, 1:2000), COL1A1 (Cat. No. AF7001, 1:1000), VEGFA (Cat. No. AF5131, 1:2000), CD31 (Cat. No. AF6191, 1:1000), PI3K (Cat. No. AF6241, 1:2000), p-PI3K (Cat. No. AF3241, 1:1000), AKT (Cat. No. AF6261, 1:1000), p-AKT (Cat. No. AF3263, 1:1000), GSK-3β (Cat. No. AF5016, 1:2000), p-GSK-3β (Cat. No. AF2016, 1:2000), β-catenin (Cat. No. 8480S, 1:1000), p-β-catenin (Cat. No. 9561S, 1:1000), and GAPDH (Cat. No. AF7021, 1:5000). Then, the membranes were washed with TBST and incubated in HRP-conjugated secondary antibodies at room temperature for 2 h. The proteins were visualized by autoradiography and analyzed with Image J software.

### Immunofluorescence

BMSCs were fixed with 4% paraformaldehyde for 30 min, permeabilized with 0.5% Triton X-100 for 15 min and blocked with 1% bovine serum albumin for 30 min, the cells were incubated with primary antibodies overnight at 4°C. Subsequently, BMSCs were washed thrice with PBS and incubated with fluorescence-conjugated secondary antibodies for 2 h, followed by incubation with DAPI for 15 min. Fluorescence images were obtained with a fluorescence microscope (BX63, Olympus, Japan).

### Angiogenesis-related assays

To further evaluate the proangiogenesis ability of POL, osteogenic differentiated BMSCs were treated with/without 1 μM POL for 7 days, the conditioned mediums were collected as previously described^22^. Whereafter, human umbilical vein endothelial cells (HUVECs, iCell Bioscience, China) were cultured in different conditions. The transwell migration assay was conducted with HUVECs (3×10^5^ cells/well) seeded into the upper chamber of 24-well transwell plates (BD Bioscience, USA). The different mediums mentioned above were added to the below chamber. After 8 h, the cells in the upper chamber were scraped away with cotton swabs. The cells that migrated to the lower chamber surface were stained with 0.1% crystal violet for 30 min. Finally, the cells were measured with an inverted microscope (IX73, Olympus, Japan). The scratch wound assay was conducted with HUVECs (2×10^5^ cells/well) seeded into 6-well plates. After confluence, the cells were scratched with a sterile yellow pipette tip and then cultured in different conditions listed above. Images of the wound were taken immediately and 24 h later. Images were analyzed by ImageJ software. The tube formation assay was conducted with HUVECs (1.5×10^4^ cells/well) seeded into matrigel precoated 96-well plates and cultured in different conditions listed above. After 8 h of culture, the tube formation in different groups were observed with the inverted microscope (IX73, Olympus, Japan).

### Surgery and treatment

A total of 48 female Sprague-Dawley rats (12-week-old, weighing 260±20 g) were gained from the Laboratory Animal Center of Southern Medical University. All the animal experiments were approved by the Animal Ethics Committee of Southern Medical University (Approval No. SMUL2023015, Date of approval: 27 March 2023). The work has been reported in accordance with the Animals in Research: Reporting In Vivo Experiments (ARRIVE) guidelines (Supplemental Digital Content 1, http://links.lww.com/JS9/D395)^23^. All rats were housed 55% humidity and 22°C constant temperature under 12 h dark/light cycles. All rats were kept warm on the healing pad (37.0±0.5°C) throughout the surgery until the rats were completely awake. A strict aseptic operation was performed during the operation, and penicillin (80 U/g) was injected to prevent infection for 3 days after surgery. After 1 week of adaptation, 30 rats were chosen for bilateral ovariectomy (OVX) surgery at random; the other 18 rats underwent the sham operation as previously described^24^. The sample size was decided based on the number of experimental groups and different time points of analysis. After 3 months, according to the method of random number table, six rats were randomly selected from each of the two groups to confirm the efficacy of OVX surgery by quantitative analysis of bone parameters. Then, all remaining rats were weighed and anesthetized via intraperitoneal injection of pentobarbital sodium (30 mg/kg; Hanlim Pharma Co., Seoul, Korea). A longitudinal approach was used to expose the right proximal tibial metaphysis. A standardized drill hole defect (3.5 mm diameter and 4.5 mm deep) was used to create a monocortical defect. After surgery, according to the method of random number table, the OVX rats were classified into two groups at random: OVX + CON group (*n*=12) and OVX + POL group (*n*=12), the sham-operated rats regarded as the Sham group (*n*=12). POL was administered intragastrically to rats in the OVX + POL group at a dose of 40 mg/kg daily. The rats in the OVX + CON group and Sham group were given normal saline by gavage. The drill-hole cortical bone defect model was chosen in this study, for animal models of drill-hole defects are easy to standardize and do not require external fixation of the bone^25^. Moreover, the monocortical bone defects induced by drill-hole injury generally take less time to heal than the osteotomy-induced bone defects^26,27^. The intervention time of POL was determined based on the previous investigation on bone repair in the tibial defect of osteoporotic rats^21^. The intervention dose of POL (40 mg/kg/d) was chosen due to this dose of POL showed a protective effect on ovariectomized rodent models^9^. After to 4 weeks of treatment, all rats underwent an X-ray radiograph, and the right tibias were collected after sacrifice. No rats died during the experiments. Six tibias were randomly chosen from each group for fluorescent labeling analysis. The other six tibias in each group were used for micro-CT scanning. After micro-CT imaging, the above six tibias were used for histology analysis.

### Sequential fluorescent labeling

All rats were injected subcutaneously with 10 mg/kg calcein (Sigma, USA) 10 and 3 days before sacrifice^28^. We prepared fluorescent agents before injection and filtered them with a 0.45 μm filter. Tibia samples in different groups were collected for hard-tissue slicing and imaged by laser confocal microscopy (FV3000, Olympus, Japan).

### Radiography and micro-CT

The bone repair was evaluated by an X-ray machine (Longsafe Imaging, Co., Ltd., China) and micro-CT (Bruker micro-CT, Kontich, Belgium). The first region of interest (ROI) of micro-CT was defined as the proximal tibial metaphysis, After 3D reconstruction, the parameters included bone mineral density (BMD), bone volume (BV), bone volume/tissue volume (BV/TV), and trabecular number (Tb.N) were measured to confirm the efficacy of OVX surgery. The second ROI was defined as the bone defect area. Measured parameters included BMD, BV/TV, Tb.N, and trabecular separation (Tb.Sp).

### Histology staining

After micro-CT imaging, the tibias were decalcified in 10% EDTA for 21 days for histology analysis. Four-μm-thick sections were applied to hematoxylin and eosin (H&E) staining and Masson’s trichrome staining. For Immunohistochemical staining, 4 μm-thick sections were incubated with primary antibodies against OCN (Cat. No. DF12303, 1:100), VEGFA (Cat. No. AF5131, 1:200), and CD31 (Cat. No. AF6191, 1:100) overnight. Then, the immune-reactivities of the sections were determined using the HRP‐streptavidin detection system. For immunofluorescence staining, 4 μm-thick sections were incubated with primary antibodies against CD31(Cat. No. AF6191, 1:200), EMCN (Cat. No. DF13357, 1:200), and β-catenin (Cat. No. 8480S, 1:100). After washed with PBS for three times, tissues were incubated with fluorescence conjugated secondary antibodies for 2 h. Subsequently, DAPI staining was carried out to stain the nuclei. The images were visualized using a BX63 microscope (Olympus, Tokyo, Japan).

### Statistical analysis

All statistical analyses were performed using SPSS software version 25.0 (IBM, New York, USA). Data were presented as mean±SEM. One-way analysis of variance (One-way ANOVA) with Tukey’s post-hoc test was utilized for multiple comparisons to determine the significance of difference. Differences were considered statistically significant when *P*<0.05.

## Results

### POL stimulated BMSCs proliferation and osteogenic differentiation

**Figure 1:**
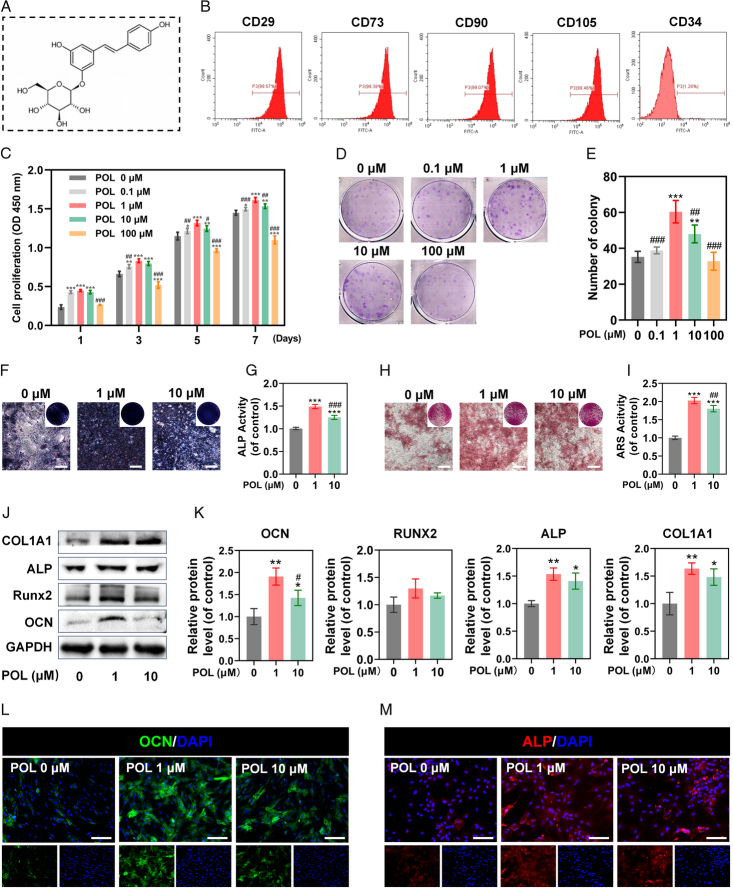
POL stimulated BMSCs proliferation and osteogenic differentiation. (A) POL chemical structure; (B) BMSCs surface markers were detected by flow cytometry; (C) The result of CCK-8 assay (*n*=5); (D, E) The result of CFU assay (*n*=5); (F) Images of ALP staining (scale bar=250 μm); (G) Quantification of ALP staining (*n*=5); (H) Images of ARS staining (scale bar=250 μm); (I) Quantification of ARS staining (*n*=5); (J, K) The protein expression levels of OCN, RUNX2, ALP, and COL1A1 were evaluated by western blot (*n*=3); (L, M) Immunofluorescence staining of OCN and ALP (scale bar=200 μm). Data were presented as mean±SEM. Compared with control group: ^*^ *P*<0.05, ^**^ *P*<0.01, ^***^ *P*<0.001. Compared with 1 μM group: ^**#**^ *P*<0.05, ^**##**^ *P*<0.01, ^**###**^ *P*<0.001.

The chemical structure of POL is presented in Figure 1A. As indicated by the flow cytometric analysis, the cells showed positive expression of CD29 (99.57%), CD73 (99.38%), CD90 (99.07%), and CD105 (99.46%), and negative expression of CD34 (1.28%) (Fig. 1B), confirming their BMSCs identity. To investigate the effect of POL on BMSCs proliferation, CCK-8 and CFU assays were firstly conducted. As per the results, both CCK-8 and CFU assays revealed that POL stimulated the BMSCs proliferation in a dose-independent manner, with the appropriate concentrations presented at 1 μM and 10 μM (Fig. 1C–E). Thus, the two concentrations were selected for the downstream assays. The osteogenesis ability of POL was then evaluated through ALP staining conducted after 7 days of osteo-induction and ARS staining after 21 days of osteo-induction. The groups of BMSCs treated with 1 μM and 10 μM POL showed higher ALP activity compared to the control group, and the optimal concentration was 1 μM (Fig. 1F,G), which was validated by ARS staining (Fig. 1H,I). To further explore how POL affects osteogenic differentiation of BMSCs, the expressions of osteogenic-specific markers were measured by qRT-PCR, western blot, and immunofluorescence staining. The expression levels of OCN, ALP, and COL1A1 were up-regulated in the POL-treated groups, but the expression levels of RUNX2 in the POL-treated groups were not significantly different compared to the control group (Fig. S1, Supplemental Digital Content 2, http://links.lww.com/JS9/D396), which was confirmed by western blot and immunofluorescence staining (Fig. 1J–M). Taken together, the above results suggested that POL could stimulated proliferation and osteogenic differentiation of BMSCs and the optimal concentration was 1 μM.

### POL promoted BMSCs-mediated angiogenesis

**Figure 2:**
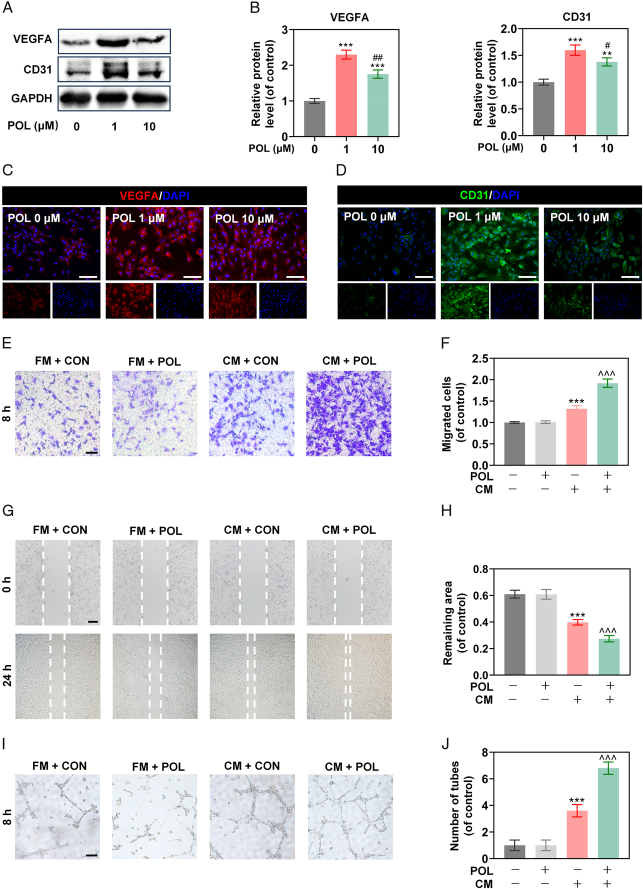
POL promoted BMSCs-mediated angiogenesis. (A, B) The protein expression levels of VEGFA and CD31 were evaluated by western blot (*n*=3); (C, D) Immunofluorescence staining of VEGFA and CD31 (scale bar=200 μm); (E) Images of transwell migration assay (scale bar=100 μm); (F) Quantification of transwell migration assay (*n*=5); (G) Images of scratch wound assay (scale bar=100 μm); (H) Quantification of scratch wound assay (*n*=5); (I) Images of tube formation assay (scale bar=100 μm); (J) Quantification of tube formation assay (*n*=5). Data were presented as mean±SEM. Compared with control group: ^**^ *P*<0.01, ^***^ *P*<0.001. Compared with 1 μm group: ^**#**^ *P*<0.05, ^**##**^ *P*<0.01. Compared with CM + CON group: **^^^** *P*<0.001.

To explore the effect of POL on angiogenesis, the expressions of angiogenic-specific markers were measured by qRT-PCR. The expression levels of VEGFA and CD31 were up-regulated in the POL-treated groups, but the expression levels of Ang-2 (angiopoietin-2) and Ang-4 (angiopoietin-4) in the POL-treated groups were not significantly different compared to the control group (Fig. S2, Supplemental Digital Content 2, http://links.lww.com/JS9/D396), which was confirmed by western blot and immunofluorescence staining (Fig. 2A–D). The results of the transwell migration assay showed that there was no significant difference in migration numbers of HUVECs between the POL-treated group and the POL-untreated group when HUVECs were cultured in fresh medium, but POL obviously increased migration numbers of HUVECs when HUVECs were cultured in conditioned medium (Fig. 2E,F), which was confirmed by scratch wound assay (Fig. 2G,H). Besides evaluating the motility of HUVECs, the capacity of angiogenesis was also determined. The results of the tube formation assay revealed that there was no significant difference in tube formation of HUVECs between the POL-treated group and the POL-untreated group when HUVECs were cultured in fresh medium, but POL obviously enhanced tube formation of HUVECs when HUVECs were cultured in conditioned medium (Fig. 2I,J). Collectively, the results mentioned above implied that instead of stimulating HUVECs angiogenesis in a direct manner, POL-promoted angiogenesis was tend to be a BMSCs-mediated manner, which suggested that the pro-osteogenesis effect of POL was coupled with its proangiogenesis effect.

### POL-induced osteogenesis-angiogenesis coupling of BMSCs via activating PI3K/AKT/GSK-3β/β-catenin signaling pathway

**Figure 3:**
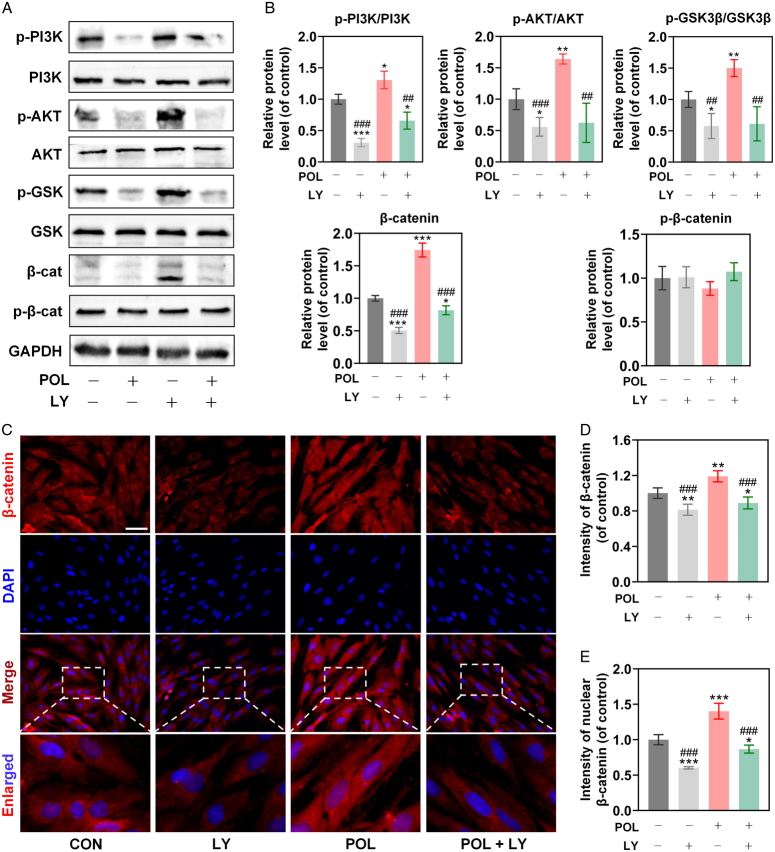
The effect of POL action was via the PI3K/AKT/GSK3-β/β-catenin pathway. (A, B) The protein expression levels of total PI3K, p-PI3K, total AKT, p-AKT, total GSK3β, p-GSK3β, total β-catenin, and p-β-catenin were evaluated by western blot (*n*=3); (C) Images of immunofluorescence staining for β-catenin (scale bar=100 μm); (D, E) Quantification of immunofluorescence staining for β-catenin (*n*=5). Data were presented as mean±SEM. Compared with control group: ^*^ *P*<0.05, ^**^ *P*<0.01, ^***^ *P*<0.001. Compared with POL group: ^**##**^ *P*<0.01, ^**###**^ *P*<0.001.

To investigate whether PI3K/AKT-mediated GSK-3β/β-catenin signaling was involved in POL-induced osteogenesis-angiogenesis coupling of BMSCs, POL with/without LY294002 (a specific inhibitor of PI3K pathway) was employed to stimulate the osteogenic BMSCs for 1 week. Then, the expression levels of total PI3K, p-PI3K, total AKT, p-AKT, total GSK3β, p-GSK3β, total β-catenin, and p-β-catenin in groups were evaluated, aiming to explore whether POL activated the PI3K/AKT/GSK-3β/β-catenin signaling pathway in the osteogenic BMSCs. Western blot analysis indicated that POL accelerated the phosphorylation of PI3K, AKT, and GSK-3β, as well as improved the expression of β-catenin. However, the positive roles of POL on PI3K/AKT/GSK-3β/β-catenin signaling was significantly reversed after cocultured with 10 μM of LY294002. Meanwhile, the inhibition of β-catenin phosphorylation by POL was also reversed after LY294002 treatment (Fig. 3A,B), which implied that PI3K/AKT was the upstream of GSK-3β/β-catenin signaling, and the PI3K/AKT/GSK-3β/β-catenin signaling was activated by POL. As shown in Figure 3C, the result of immunofluorescence staining indicated that POL enforced expression of β-catenin (red) both in the cytoplasm and nuclei (blue), while LY294002 reversed the expression as well as the β-catenin translocation, which was confirmed by the quantitative analysis of β-catenin immunofluorescence (Fig. 3D,E). Collectively, these results indicated that the PI3K/AKT/GSK-3β/β-catenin signaling pathway was involved in the POL-treated BMSCs.

**Figure 4:**
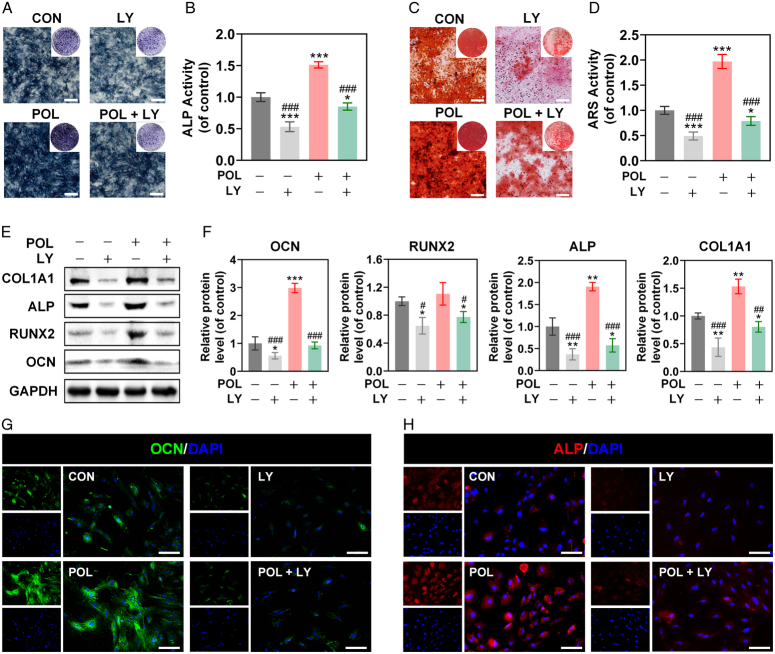
Inhibition of PI3K/AKT/GSK-3β/β-catenin signaling pathway reduced the effect of POL on pro-osteogenesis. (A) Images of ALP staining (scale bar=250 μm); (B) Quantification of ALP staining (*n*=5); (C) Images of ARS staining (scale bar=250 μm); (D) Quantification of ARS staining (*n*=5); (E-F) LY294002 reversed the proexpression effect of POL on OCN, RUNX2, ALP, and COL1A1 were evaluated by western blot (*n*=3); (G-H) LY294002 reversed the proexpression effect of POL on OCN and ALP were evaluated by immunofluorescence staining (scale bar=100 μm). Data were presented as mean±SEM. Compared with control group: ^*^ *P*<0.05, ^**^ *P*<0.01, ^***^ *P*<0.001. Compared with POL group: ^**#**^ *P*<0.05, ^**##**^ *P*<0.01, ^**###**^ *P*<0.001.

To detect the role of PI3K/AKT/GSK-3β/β-catenin signaling pathway in the POL-induced osteogenic differentiation, a serial of osteogenesis-related assays were subsequently carried out. The results of ALP and ARS staining presented the increased ALP activity and calcium nodules deposition in the POL group, while these effects were significantly attenuated when co-cultured with LY294002 (Fig. 4A–D). Western blot analysis confirmed that LY294002 reversed the expression levels of OCN, ALP, and COL1A1, which were elevated by POL (Fig. 4E,F). Subsequently, the results of immunofluorescence staining confirmed the positive role of the PI3K/AKT/GSK3-β/β-catenin pathway in the process of POL-induced osteogenesis (Fig. 4G,H). The above results suggested that POL promoted osteogenesis of BMSCs via activating PI3K/AKT/GSK-3β/β-catenin signaling pathway.

**Figure 5:**
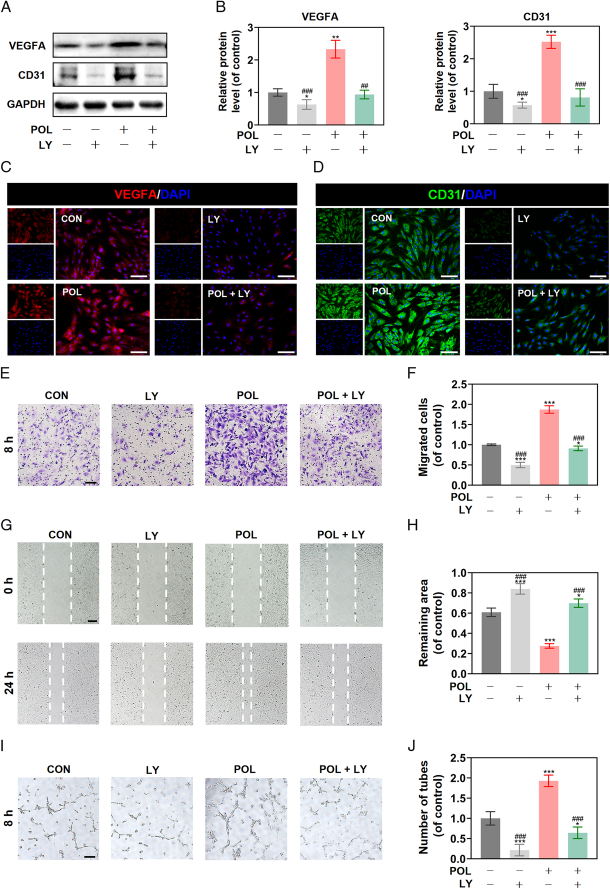
Inhibition of PI3K/AKT/GSK-3β/β-catenin signaling pathway reduced the effect of POL on inducing osteogenesis-angiogenesis coupling. (A, B) LY294002 reversed the proexpression effect of POL on VEGFA and CD31 were evaluated by western blot (*n*=3); (C, D) LY294002 reversed the proexpression effect of POL on VEGFA and CD31 were evaluated by immunofluorescence staining (scale bar=200 μm); (E) Images of transwell migration assay (scale bar=100 μm); (F) Quantification of transwell migration assay (*n*=5); (G) Images of scratch wound assay (scale bar=100 μm); (H) Quantification of scratch wound assay (*n*=5); (I) Images of tube formation assay (scale bar=100 μm); (J) Quantification of tube formation assay (*n*=5). Data were presented as mean±SEM. Compared with control group: ^*^ *P*<0.05, ^**^ *P*<0.01, ^***^ *P*<0.001. Compared with POL group: ^**##**^ *P*<0.01, ^**###**^ *P*<0.001.

Next, we explored whether the PI3K/AKT/GSK-3β/β-catenin signaling pathway was also involved in the POL-mediated angiogenesis of BMSCs. Western blot analysis showed that LY294002 reversed the expression levels of VEGFA and CD31, which were upregulated by POL (Fig. 5A,B). Consistent with that, the results of immunofluorescence staining confirmed the positive role of the PI3K/AKT/GSK3-β/β-catenin pathway in the process of POL-induced angiogenesis (Fig. 5C,D). Furthermore, the migration and tube formation capacity were evaluated by angiogenesis-related assays, which revealed that POL notably enhanced the effect of BMSCs-mediated proangiogenesis, while LY294002 attenuated this effect (Fig. 5E–J). Together, these findings confirmed that the PI3K/AKT/GSK3-β/β-catenin pathway was involved in the POL action on osteogenesis-angiogenesis coupling of BMSCs.

### POL enhanced the capacity of osteoporotic bone repair by stimulating osteogenesis

**Figure 6:**
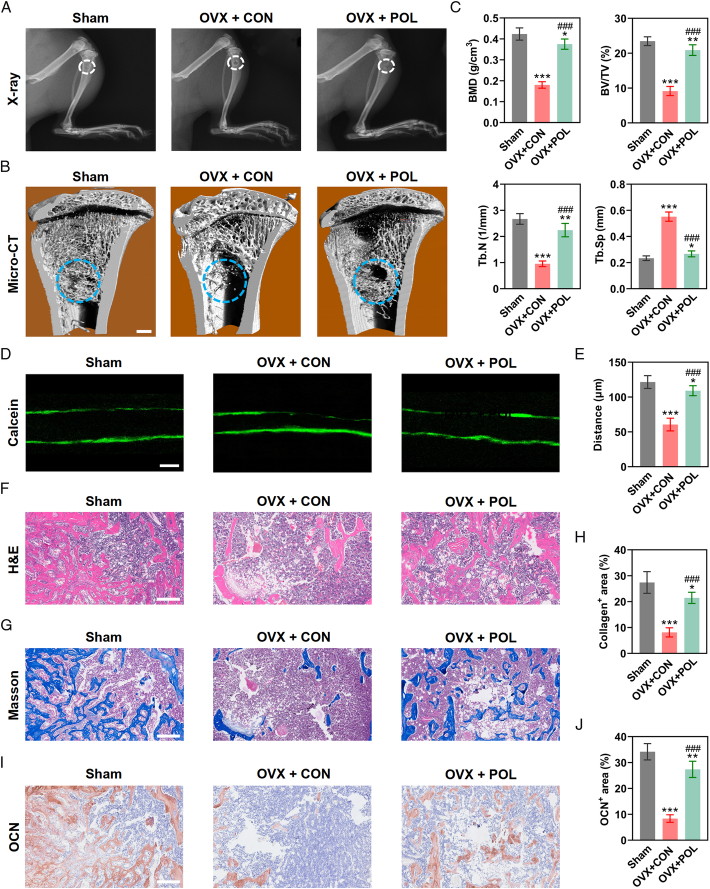
POL enhanced the capacity of osteoporotic bone repair by stimulating osteogenesis. (A) The digitized images of X-radiograph; (B) The 3D reconstruction images of micro-CT (scale bar=1 mm); (C) Micro-CT analyses of BMD, BV/TV, Tb.N, and Tb.Sp (*n*=6); (D) Images of sequential fluorescent labeling (scale bar=100 μm); (E) Quantification of sequential fluorescent labeling (*n*=6); (F, G) Histological evaluation of the bone defect area by H&E staining and Masson’s trichrome staining (scale bar=200 μm); (H) Quantification of collagen (*n*=6); (I) Images of immunohistochemical staining for OCN (scale bar=200 μm); (J) Quantification of immunohistochemical staining for OCN (*n*=6). Data were presented as mean±SEM. Compared with Sham group: ^*^ *P*<0.05, ^**^ *P*<0.01, ^***^ *P*<0.001. Compared with OVX + CON group: ^**###**^ *P*<0.001.

After confirmation of osteoporosis (Fig. S3, Supplemental Digital Content 2, http://links.lww.com/JS9/D396), the rats underwent the tibial defect operation and received POL treatment for 4 weeks. Both the digitized images of the X-radiograph and 3D reconstruction images of micro-CT exhibited that the area of bone regeneration enlarged more observably in the OVX + POL group than those in the OVX + CON group (Fig. 6A,B). Accordingly, further analysis of micro-CT showed that the values of BMD, BV/TV, and Tb.N in the OVX + CON group were the lowest, and the value of Tb.Sp in the OVX + CON group was highest among all groups (Fig. 6C). Histologically, bone mineralization labeled by calcein revealed that the distance strip (green) in the OVX + POL group was wider compared to the OVX + CON group (Fig. 6D,E). Meanwhile, H&E staining showed the volume of cancellous bone in the OVX + POL group was higher than the OVX+ CON group, but still lower than the Sham group (Fig. 6F), which was consistent with the result of micro-CT. Finally, the Masson’s trichrome staining and the immunohistochemical staining indicated that POL treatment obviously improved collagen formation and bone mineralization (Fig. 6G–J). Therefore, the above-mentioned results exhibited that POL enhanced the capacity of osteoporotic bone repair by stimulating osteogenesis.

### POL accelerated osteoporotic bone repair by stimulating osteogenesis and angiogenesis via up-regulating β-catenin expression

**Figure 7:**
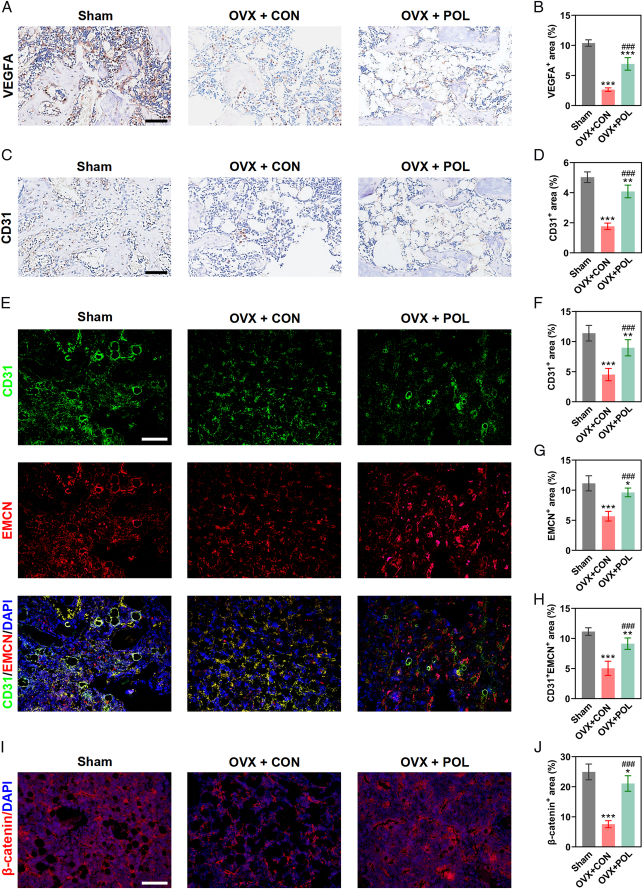
POL accelerated osteoporotic bone repair by stimulating osteogenesis and angiogenesis via up-regulating β-catenin expression. (A) Images of immunohistochemical staining for VEGFA (scale bar=200 μm); (B) Quantification of immunohistochemical staining for VEGFA (*n*=6); (C) Images of immunohistochemical staining for CD31 (scale bar=200 μm); (D) Quantification of immunohistochemical staining for CD31 (*n*=6); (E) Images of CD31 and EMCN immunofluorescence double-staining (scale bar=100 μm); (F–H) Quantification of CD31 and EMCN immunofluorescence double-staining (*n*=6); (I) Images of immunofluorescence staining for β-catenin (scale bar=100 μm); (J) Quantification of immunofluorescence staining for β-catenin (*n*=6). Data were presented as mean±SEM. Compared with Sham group: ^*^ *P*<0.05, ^**^ *P*<0.01, ^***^ *P*<0.001. Compared with OVX + CON group: ^**###**^ *P*<0.001.

Immunohistochemical staining of the angiogenic-specific markers (VEGFA and CD31) exposed more positive areas in the OVX + POL group than those in the OVX + CON group (Fig. 7A–D). Furthermore, the CD31^hi^EMCN^hi^ vessels in bone defect area were observed. CD31 and EMCN immunofluorescence double-staining revealed a greater population of CD31^hi^EMCN^hi^ cells within the callus in the OVX + POL group compared to the OVX + CON group (Fig. 7E–H). Subsequently, immunofluorescence staining showed a higher expression level of β-catenin in the OVX + POL group than in the OVX + CON group (Fig. 7I,J). Collectively, the above results suggested that POL accelerated osteoporotic bone repair by stimulating osteogenesis and angiogenesis via up-regulating β-catenin expression.

## Discussion

As far as we know, this study presented the first evidence that POL could induce osteogenesis-angiogenesis coupling. Further study revealed that POL could accelerate osteoporotic bone repair by inducing the osteogenesis-angiogenesis coupling of BMSCs via activating PI3K/AKT/GSK-3β/β-catenin signaling pathway. The osteogenic BMSCs not only accelerate the calcium deposition and collagen synthesis, but also promote the angiogenesis of endothelial cells through secreting numerous cytokines and growth factors^29^. In return, the enhanced angiogenesis, especially the newly formed type H vessels, which highly expresses CD31 and endomucin (CD31^hi^EMCN^hi^), boosts the BMSCs-mediated bone regeneration by delivering oxygen, nutrients, hormones, and growth factors^30^. In recent years, more and more researches revealed that angiogenesis as well as osteogenesis was also a crucial mechanism for osteoporotic bone repair^31,32^. And moreover, when bone defect occurs in patient with osteoporosis, the treatment of bone defect become more difficult due to disordered angiogenesis^33^. Thus, it is very significant to explore the role of POL on angiogenesis during osteoporotic bone repair.

Bone is a highly vascularized tissue, in which vascularization and mineralization are concurrent processes during bone repair^34,35^. Both osteogenesis and angiogenesis are closely related to bone repair^36^. Therefore, it is essential to focus on angiogenesis when studying bone repair. To date, there are no reported research results of POL on angiogenesis during bone repair. In this research, we first identified the pro-osteogenesis of POL, which was verified by the previous study^9^, and further research found that POL elevated the expression levels of angiogenic-specific markers (VEGFA and CD31), which implied that POL may possess the ability to promote angiogenesis. Previous research has revealed that osteogenesis and angiogenesis are closely linked because of the cross-talk between mesenchymal stem cells and endothelial cells (ECs)^37^. VEGFA has been proved to play an intermediary role in the interaction between BMSCs and ECs^38^. In addition, of specific interest here is that the optimal concentration of POL for enhancing expression of osteogenic-specific and angiogenic-specific markers was consistent. Based on that, we speculated that the pro-osteogenesis effect of POL may be closely related to its proangiogenesis effect.

To test the above conjecture, we investigated the relationship between osteogenesis and angiogenesis of POL by constructing the indirect co-culture system of BMSCs and HUVECs as previously described^22^. What was not so encouraging, however, was the fact that POL could not increase migration numbers and enhance tube formation of HUVECs when HUVECs were cultured in fresh medium. It is interesting, though, that POL increased migration numbers and enhanced tube formation of HUVECs when HUVECs were cultured in a conditioned medium. The above results indicated that POL could not promote angiogenesis in a direct manner, but POL possessed the ability to promote BMSCs-mediated angiogenesis, which suggested that the pro-osteogenesis effect of POL was coupled with its proangiogenesis effect. Further research found that the PI3K/AKT/GSK-3β/β-catenin signaling pathway was involved in the process of POL-induced osteogenesis and angiogenesis. Subsequent experiments revealed that POL notably enhanced the effect of BMSCs-mediated proangiogenesis, while LY294002 attenuated this effect, which confirmed that the PI3K/AKT/GSK3-β/β-catenin pathway was involved in the POL action on osteogenesis-angiogenesis coupling of BMSCs.

**Figure 8:**
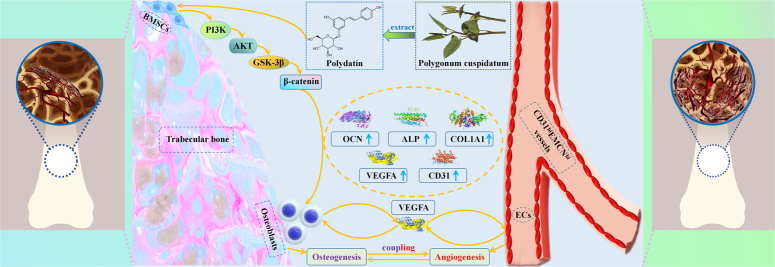
The potential mechanism of POL on osteoporotic bone repair.

After that, using the osteoporotic bone defect rat model, we found that POL could improve bone-repairing capacity by stimulating osteogenesis in osteoporotic bone defect rats. Because of the pro-osteogenesis effect of POL was coupled with its proangiogenesis effect, we next determined the role of POL on angiogenesis during osteoporotic bone repair. The results of immunohistochemical staining showed that POL enhanced the expression of VEGFA and CD31 in the bone defect area. The result of CD31 and EMCN immunofluorescence double-staining revealed that the number of type H vessels were increased in the OVX + POL group than in the OVX + CON group. Type H vessels have been shown to couple osteogenesis with angiogenesis^39–41^, which implied that POL-induced the osteogenesis-angiogenesis coupling in vivo. Single and double fluorochrome labeling, a direct histologic marker of bone formation. The distance between double labels was wider in the Sham group than in the OVX + CON group, which indicated that osteoporosis reduced the rate of bone formation in the defect area. The distance between double labels was wider in the OVX + POL group than in the OVX + CON group, which confirmed that POL accelerated the rate of bone formation in the defect area. Comprehensive analysis of results in vitro and in vivo, we found that POL accelerated osteoporotic bone repair by inducing the osteogenesis-angiogenesis coupling of BMSCs, the potential mechanism was shown in Figure 8.

Although the therapeutic dose of POL (40 mg/kg/d) was shown to be an effective dose for the treatment of osteoporotic bone defect in OVX rats, however, the therapeutic human dosage remains to be further studied. In addition, mesenchymal stem cells-derived extracellular vesicles, as inherent cell-secreted natural products, have emerged as promising treatments for bone-related diseases^42,43^. In contrast to generic extracellular vesicles, mesenchymal stem cells-derived extracellular vesicles offer distinct advantages, rendering them exceptional drug carriers, which have shown potential as drug transport platforms for targeted delivery of exogenous cargo to specific cell types or tissues^44–46^. Thus, we will explore the therapeutic potency of mesenchymal stem cells-derived extracellular vesicles as drug vehicles to deliver POL to defect area in the future study, which is expected to be a novel strategy for osteoporotic bone repair.

## Conclusion

The study highlighted the importance of the effective coupling of osteogenesis and angiogenesis, especially for osteoporotic bone repair, which revealed that POL could accelerate osteoporotic bone repair by inducing the osteogenesis-angiogenesis coupling of BMSCs via activating PI3K/AKT/GSK-3β/β-catenin signaling pathway, which provided new insight and strategy for osteoporotic bone repair.

## Ethical approval

All the animal experiments were approved by the Animal Ethics Committee of Southern Medical University (Approval No. SMUL2023015, Date of approval: 27 March 2023, License No. of SCXK (YUE) 2021-0041, Guangzhou, China).

## Consent

Not applicable.

## Source of funding

This work was supported by the China Postdoctoral Science Foundation (No. 2024M751344) and the Postdoctoral Fellowship Program of China Postdoctoral Science Foundation (No. GZC20231088).

## Author contribution

C.Z.: conceptualization, formal analysis, investigation, methodology, writing – original draft, and writing – review and editing; G.H. and Y.L.: data curation, formal analysis, investigation, and validation; S.Z.: conceptualization, formal analysis, funding acquisition, investigation, methodology, project administration, supervision, validation, writing – original draft, and Writing – review and editing.

## Conflicts of interest disclosure

All authors declare no conflicts of interest.

## Research registration unique identifying number (UIN)

Not applicable.

## Guarantor

Sheng Zheng.

## Data availability statement

The datasets used and analyzed during the current study are available from the corresponding author on reasonable request.

## Provenance and peer review

Not commissioned, externally peer-reviewed.

## Supplementary Material
