# Supplementary material for: Polydatin accelerates osteoporotic bone repair by inducing the osteogenesis-angiogenesis coupling of bone marrow mesenchymal stem cells via the PI3K/AKT/GSK-3β/β-catenin pathway
Source: Int J Surg. 2024 Sep 6;111(1):411–25. doi: 10.1097/JS9.0000000000002075 (PMC11745762; doi:10.1097/JS9.0000000000002075)
Supplement: Supplementary file 2 [file js9-111-0411-s002.docx]

**Supplementary Item**

**Supplementary Table**

**Table S1**

**Real-time PCR primer sequences used in the study.**

| **Gene** | **Forward primer** | **Reverse primer** |
| --- | --- | --- |
| OCN | GTCAGACTACAACATCCAGAAG | CGAGTATCTTCCTGTTTGACC |
| RUNX2 | GAGCGTTCAACGGCACAG | GACAGTAGACTCCACGACA |
| ALP | ACCATTCCCACGTCTTCACATTT | AGACATTCTCTCGTTCACCGCC |
| COL1A1 | TGTCGTTCAACGGCACAG | TGTGGTAGACTCCACGACA |
| VEGFA | TCAGGAGGACCTTGTGTGATC  AG | CATTGCTCTGTACCTTGGGAA |
| CD31 | CACCGTGATACTGAACAGCAA | GTCACAATCCCACCTTCTGTC |
| Ang-2 | GAAGAAGGAGATGGTGGAGA | CGTCTGGTTGAGCAAACTG |
| Ang-4 | GCTCCTCAGGGCACCAAGTTC | CACAGGCGTCAAACCACCAC |
| GAPDH | GGCATGGACTGTGGTCATGAG | TGCACCACCAACTGTTAGC |

**Supplementary Figures**

**
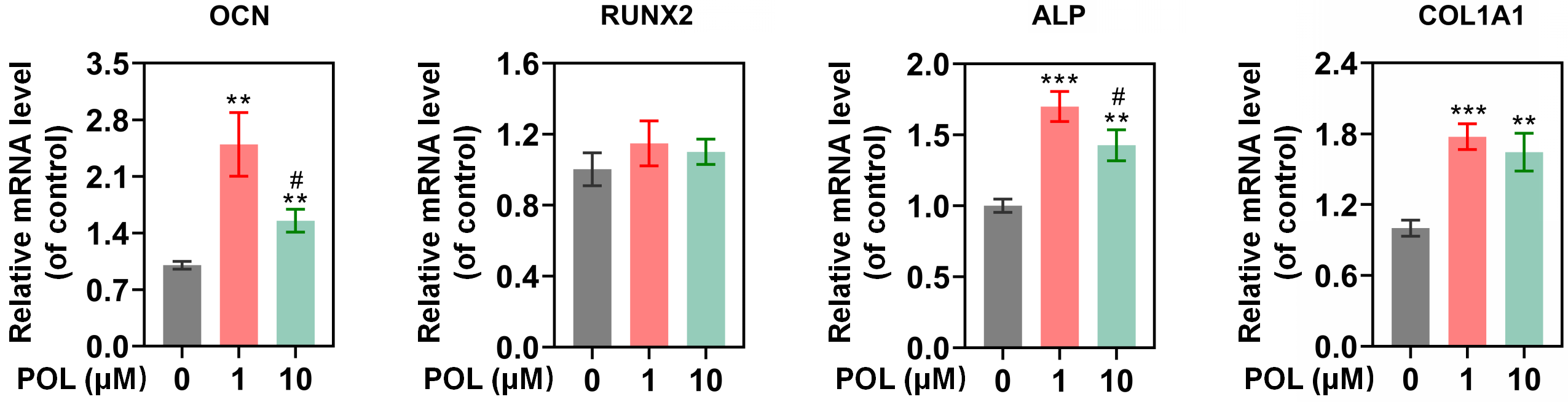
**

**F****igure S1.** The gene expression levels of osteogenic-specific markers (OCN, RUNX2, ALP, and COL1A1) were evaluated by qRT-PCR (n = 3). Data were presented as mean ± SEM. Compared with control group: **^**^***P* < 0.01, **^**^****^*^***P* < 0.001. Compared with 1 μM group: **^#^***P* < 0.05.


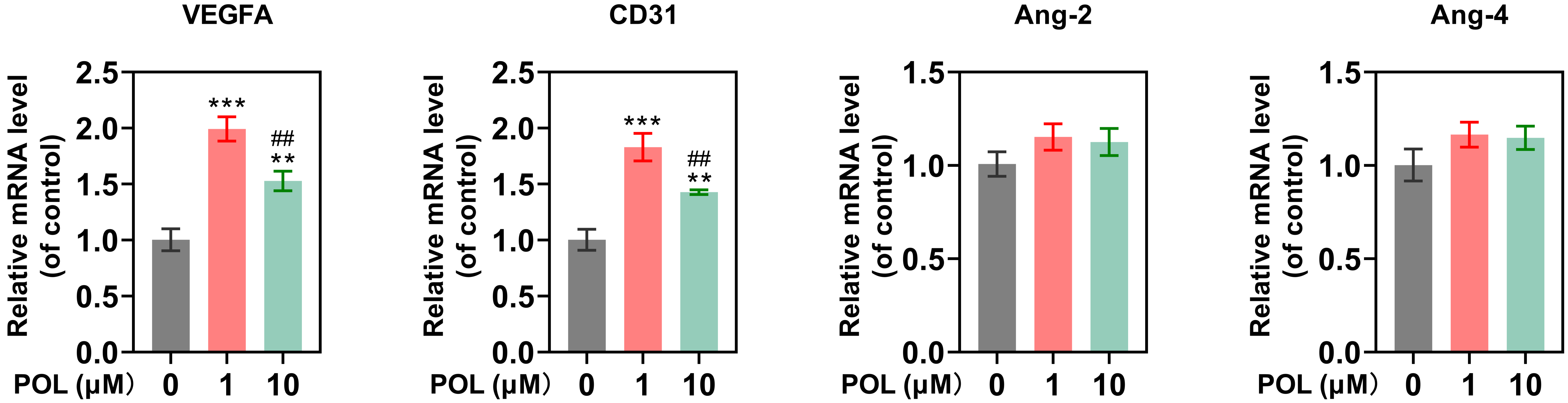


**Figure S2.** The gene expression levels of angiogenic-specific markers (VEGFA, CD31, Ang-2, and Ang-4) were evaluated by qRT-PCR (n = 3). Data were presented as mean ± SEM. Compared with control group: **^**^***P* < 0.01, **^***^***P* < 0.001. Compared with 1 μM group: **^##^***P* < 0.01.


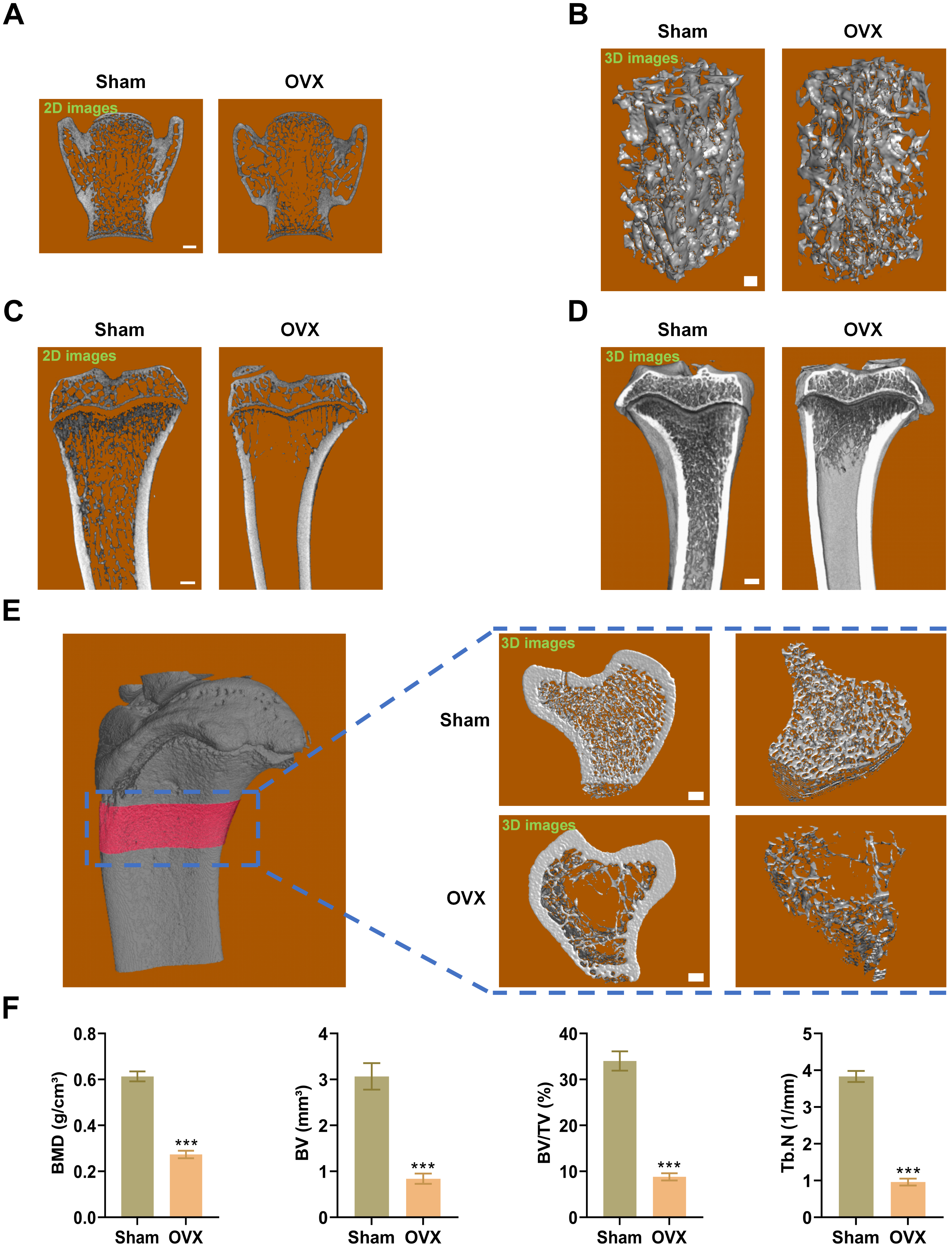


**Figure S3.** Confirmation of osteoporosis model. (A-B) The 2D scanned and 3D stereogram images of the vertebras in two groups (scale bar = 1 mm); (C-D) The 2D scanned and 3D sectioned images of the tibias in two groups (scale bar = 1 mm); (E) The 3D constructed images of the proximal metaphysis of the tibias in two groups (scale bar = 1 mm); (F) Micro-CT analyses of BMD, BV, BV/TV, and Tb.N (n = 6). Data were presented as mean ± SEM. Compared with Sham group: **^***^***P* < 0.001.
